# Decreased circulating clusterin reflects severe liver complications after hepatoportoenterostomy of biliary atresia

**DOI:** 10.1038/s41598-020-76875-9

**Published:** 2020-11-12

**Authors:** Wanvisa Udomsinprasert, Yong Poovorawan, Voranush Chongsrisawat, Paisarn Vejchapipat, Sittisak Honsawek

**Affiliations:** 1 Department of Biochemistry, Faculty of Pharmacy, Mahidol University, 447 Sri-Ayudthaya Road, Rajathevi, Bangkok, 10400 Thailand; 2 Center of Excellence in Clinical Virology, Department of Pediatrics, Faculty of Medicine, King Chulalongkorn Memorial Hospital, Chulalongkorn University, Bangkok, 10330 Thailand; 3 Department of Surgery, Faculty of Medicine, King Chulalongkorn Memorial Hospital, Thai Red Cross Society, Chulalongkorn University, Bangkok, 10330 Thailand; 4 Osteoarthritis and Musculoskeleton Research Unit, Department of Biochemistry, Faculty of Medicine, King Chulalongkorn Memorial Hospital, Thai Red Cross Society, Chulalongkorn University, Bangkok, 10330 Thailand

**Keywords:** Biomarkers, Medical research

## Abstract

This study aimed to determine whether circulating levels of clusterin (CLU), an extracellular chaperone implicated in cholestatic and fibrotic processes, are associated with clinical parameters of post-operative BA patients and could serve as a BA biomarker. Ninety-six BA patients and 56 healthy controls were recruited. Circulating CLU levels were measured using enzyme-linked immunosorbent assay. Circulating CLU levels were significantly reduced in BA patients – especially those with worse outcomes including jaundice, severe liver fibrosis, and late-stage of hepatic dysfunction. Multivariate linear regression analysis revealed that circulating CLU levels were negatively associated with outcome parameters indicating jaundice status, degree of fibrosis, and liver dysfunction, but positively correlated with serum albumin and platelet number of BA patients. Lower circulating CLU levels were considerably associated with poor survival of post-operative BA patients. Receiver-operating characteristic curve analysis demonstrated a diagnostic value of circulating CLU as a non-invasive indicator for poor outcomes of BA patients (AUC = 0.85), with a sensitivity of 81.5% and a specificity of 73.5%. All findings indicate that reduced circulating CLU might reflect poor outcomes of BA patients and have potential as a novel biomarker for the disease severity following Kasai-operation.

## Introduction

Biliary atresia (BA) is a rare cholestatic liver disease of neonates characterized by obstruction of the biliary system, leading to progressive hepatic fibrosis, cirrhosis, and eventually to end-stage liver disease^1^. Despite advances in understanding of causative factors involved in BA etiology, its exact causes are not yet clearly defined. Given the unclarified pathogenesis and delayed diagnosis, the current treatment being hepatoportoenterostomy (HPE, also known as Kasai operation) could not stop progression of severe liver complications in some cases^2^. As such, most BA patients will undergo liver transplantation^3^. Despite refined usage of conventional liver biomarkers including serum enzyme activities of aminotransferases and bilirubin levels, alterations in levels of those indicators are not reportedly specific for BA pathogenesis and are inadequate for early identification of BA^4^. For the above-mentioned reasons, it would seem more appropriate to identify specific biomarkers for early BA progression, which would be useful for improving HPE outcomes and slowing down the need for pediatric liver transplantation.

Of various molecules known to be involved in cholestasis and liver fibrosis, clusterin (CLU) exerting its extracellular chaperone-like activity implicated in homeostasis/proteostasis of extracellular proteins is gaining increasing interest as a possible mediator for fibrogenesis. As to its general roles, CLU, a secreted heterodimeric glycoprotein ubiquitously expressed in a variety of human tissues and body fluids^5^, shares functional similarities with small heat shock proteins that inhibit aggregation and precipitation of secreted proteins and assist with clearance of cellular debris from the extracellular matrix (ECM) via endocytosis and lysosomal degradation^6,7^. Generally, secretory CLU, a predominant isoform of CLU widely found in most body fluids of humans, has been functionally involved in a wide range of biological processes including cell apoptosis, cell adhesion, tissue remodeling, and cell cycle regulation, in addition to lipid transportation^8–12^. On the basis of its property, alterations in CLU expression have been reportedly associated with poor prognosis in cancer patients – especially in patients with hepatocellular carcinoma (HCC)^13,14^. Besides its overexpression in HCC, CLU expression was detected in the bile plugs in all cholestatic liver diseases and related to fibrotic areas of the liver with its co-localization in the elastic fibers^15^, thereby highlighting the possible relevance of CLU to cholestatic and fibrotic processes in the liver. From this premise, CLU could be possibly developed as a novel biomarker for severe liver complications of post-operative BA patients—especially progressive liver fibrosis.

Although CLU expression in the liver and the biliary tracts of patients with cholestatic liver diseases has been thoroughly explored, until now no attempt has been made to capture the breadth of systemic production of CLU related to BA outcomes—particularly liver fibrosis. Accordingly, the present study aimed to evaluate circulating CLU levels in post-operative BA patients compared to healthy controls and to determine whether circulating CLU levels were associated with outcome parameters of the patients.

## Materials and methods

The study protocol conformed to the ethical standards outlined in the Declaration of Helsinki and was approved by the Institutional Review Board of the Faculty of Medicine, Chulalongkorn University (IRB number 356/52) and the Faculty of Dentistry/Faculty of Pharmacy, Mahidol University (IRB number 2019/026.2015). Written informed consent was obtained from the participants’ parents.

### Study subjects

This case–control study consisted of 96 post-operative BA patients and 56 age-matched unaffected volunteers. All BA patients were diagnosed by intraoperative cholangiography and were surgically treated with original Kasai operation. None of the BA patients in the study had undergone liver transplantation or exhibited signs and symptoms of fever or ascending cholangitis at the time of blood sampling. Healthy controls who attended the Well Baby Clinic at our institution for vaccination had normal physical findings and no underlying disease. In terms of bile flow establishment, BA patients were stratified with regard to serum total bilirubin (TB) into non-jaundice (TB < 2 mg/dL, *n* = 61) and persistent jaundice groups (TB ≥ 2 mg/dL, *n* = 35). According to severity of liver fibrosis (liver stiffness values), the patients were also classified into mild fibrosis (F0–F2: 0–9.7 kPa, *n* = 28) and severe fibrosis groups (F3–F4: > 9.7 kPa, *n* = 68). Based on alanine aminotransferase (ALT) values indicating severity of hepatic injury, BA patients were divided into early-stage (ALT < 100 IU/L, *n* = 46) and late-stage groups (ALT ≥ 100 IU/L, *n* = 50). Regarding their jaundice and severe fibrosis statuses, the patients were categorized into good outcome (TB < 2 mg/dL, F0–F2: 0–9.7 kPa, *n* = 27) and poor outcome groups (TB ≥ 2 mg/dL, F3–F4: > 9.7 kPa, *n* = 34), based on their values of TB and liver stiffness.

### Clinical assessments of outcomes

After overnight fast, peripheral venous blood samples were drawn from healthy controls and BA patients during their annual routine follow-up in ethylenediaminetetraacetic acid and clot blood tubes for routine laboratory tests including aspartate aminotransferase (AST), ALT, alkaline phosphatase (ALP), serum albumin, TB, direct bilirubin (DB), platelet number, prothrombin time (PT), partial thromboplastin time (PTT), AST to platelet ratio index (APRI), and international normalized ratio (INR). All of the immediately aforementioned tests were performed on a Roche Hitachi 912 chemistry analyzer (Roche Diagnostics, Basel, Switzerland). Measurement of liver stiffness by transient elastography was performed using a Fibroscan (EchoSens, Paris, France).

### Quantitation of circulating CLU levels

Circulating CLU levels were measured in duplicate for each sample using a commercial sandwich enzyme-linked immunosorbent assay (ELISA) kit (R&D Systems, Minneapolis, MN, United States), according to manufacturer’s instructions.

### Statistical analysis

All statistical analyses were performed using SPSS Statistics version 22.0 (SPSS Inc., Chicago, IL, USA). Comparisons between means were evaluated by Student’s *t*-test (for 2 groups) and one-way analysis of variance (ANOVA, > 2 groups) with a Tukey post hoc test, while Mann–Whitney *U* test and Kruskal–Wallis *H* test were employed for comparisons of abnormally distributed continuous variables. Correlations between circulating CLU levels and clinical parameters were assessed using Spearman’s rho correlation coefficient (*r*). Multivariate logistic regression models were employed to determine the roles of confounding factors. The survival curves were drawn, according to the Kaplan–Meier analysis with end points of death, in which the differences of survival curves were determined using log-rank test. Receiver operating characteristic (ROC) curve and the area under the ROC curve (AUC) were calculated to assess the feasibility of using circulating CLU as a possible biomarker for post-operative BA. Data are presented as mean ± standard deviation (SD). For all statistics, a *P*-value less than 0.05 (based on a two-tailed test) was considered statistically significant.

## Results

### Clinical characteristics of study participants

Baseline demographic and clinical characteristics of 96 post-Kasai BA patients and 56 healthy volunteers are summarized in Table 1. There were no significant differences in mean age and gender ratio between BA patients and healthy controls. As expected, BA patients had substantially higher liver stiffness, AST, and ALT values than age-matched healthy controls (*P* < 0.001).

**Table 1:** Demographic and clinical characteristics of healthy controls and BA patients.

Variables	Healthy controls (*n* = 56)	BA patients (*n* = 96)	*P*-value
Gender (male:female)	32:24	50:46	0.55
Age (years)	8.92 ± 0.47	10.00 ± 6.17	0.59
BMI (kg/m^2^)	15.78 ± 2.19	17.23 ± 3.07	0.53
Liver stiffness (kPa)	4.05 ± 0.11	31.02 ± 2.38	< 0.001*
AST (IU/L)	27.12 ± 0.89	133.72 ± 9.90	< 0.001*
ALT (IU/L)	9.16 ± 0.75	127.04 ± 9.12	< 0.001*
ALP (IU/L)	–	425.85 ± 34.05	NA
Albumin (g/dL)	–	4.07 ± 0.84	NA
TB (mg/dL)	–	2.01 ± 1.41	NA
DB (mg/dL)	–	1.70 ± 1.32	NA
Platelet (cells/m^3^)	–	162,409.00 ± 94,692.86	NA
PT (s)	–	12.44 ± 1.32	NA
PTT (s)	–	29.83 ± 2.88	NA
APRI	–	2.87 ± 2.07	NA
INR	–	1.09 ± 0.12	NA

*Difference is considered statistically significant at *P*-value less than 0.05 (two-tailed).

*ALP* alkaline phosphatase, *ALT* alanine aminotransferase, *APRI* AST to platelet ratio index, *AST* aspartate aminotransferase, *BA* biliary atresia, *BMI* body mass index, *DB* direct bilirubin, *INR* international normalized ratio, *PT* prothrombin time, *PTT* partial thromboplastin time, *TB* total bilirubin, *NA* not available.

### Circulating CLU levels in BA subjects with different subgroups

**Figure 1:**
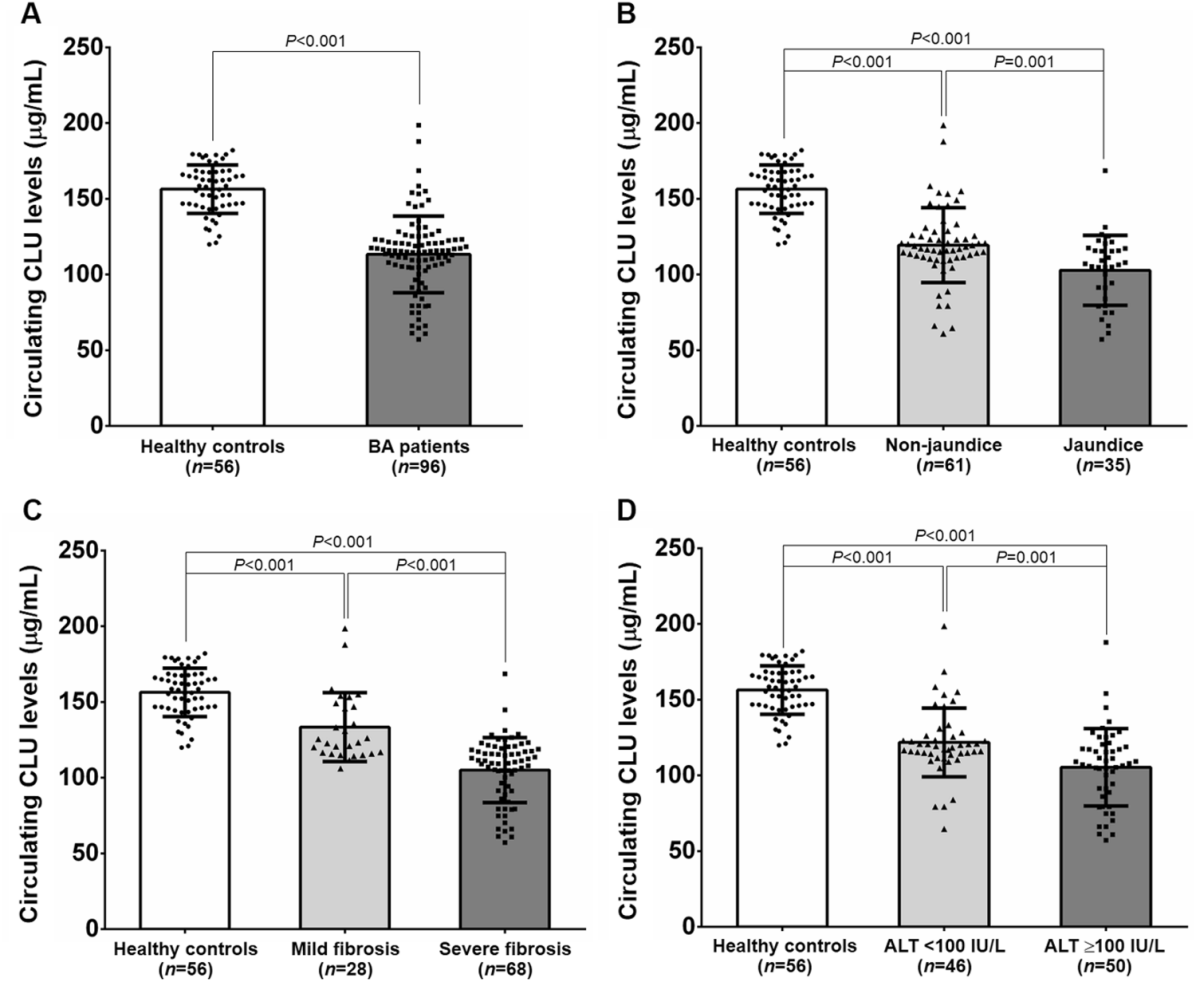
Circulating CLU levels in subjects among different groups. **(A)** Healthy controls and BA patients. **(B)** BA subgroups including non-jaundice and jaundice. **(C)** BA patients with mild fibrosis (F0–F2) and severe fibrosis (F3–F4). **(D)** BA patients according to severity of hepatic damage (ALT values).

Mean circulating CLU levels of BA patients were significantly lower than those of healthy controls (*P* < 0.001) (Fig. 1A). In stratified analysis according to jaundice status, BA patients with persistent jaundice showed significantly reduced circulating CLU levels, compared with those with non-jaundice and healthy controls (*P* = 0.001, *P* < 0.001, respectively) (Fig. 1B). In conjunction with this, circulating CLU levels were remarkably lower in BA patients without jaundice than those in healthy controls (*P* < 0.001). When severity of liver fibrosis was considered, circulating CLU levels were significantly decreased in BA patients with severe fibrosis, compared with those with mild fibrosis and healthy controls (*P* < 0.001, *P* < 0.001, respectively). Similarly, BA patients with mild fibrosis exhibited considerably lower circulating CLU levels than healthy controls (*P* < 0.001) (Fig. 1C). With regard to ALT values reflecting severity of hepatic injury, circulating CLU levels were found to be significantly declined in BA patients with high ALT values, compared with the patients with low ALT values and healthy controls (*P* = 0.001, *P* < 0.001, respectively). Correspondingly, BA patients with low ALT values had significantly reduced circulating CLU levels, compared to healthy controls (*P* < 0.001) (Fig. 1D).

### Associations between circulating CLU levels and clinical parameters

Subsequently, we determined whether circulating CLU levels were associated with clinical variables of BA patients. Spearman’s rho correlation analysis showed a significantly negative correlation of circulating CLU levels with outcome parameters including liver stiffness (*r* = − 0.54, *P* < 0.001), AST (*r* = − 0.44, *P* < 0.001), ALT (*r* = − 0.39, *P* < 0.001), ALP (*r* = − 0.37, *P* < 0.001), TB (*r* = − 0.45, *P* < 0.001), DB (*r* = − 0.55, *P* < 0.001), APRI (*r* = **− **0.46, *P* < 0.001), and INR values (*r* = − 0.27, *P* = 0.035) in BA patients. Instead, circulating CLU levels were observed to be positively related to serum albumin (*r* = 0.42, *P* < 0.001) and platelet number (*r* = 0.31, *P* = 0.013) in the patients (Table 2). To further verify the independent associations of clinical parameters with circulating CLU levels in BA patients, we conducted multivariate linear regression analysis. After adjustments for confounding variables including age, gender, and BMI, a decrease in circulating CLU levels was significantly associated with raised values of liver stiffness (β-coefficient = − 0.50; 95% CI: − 0.71 to − 0.30; *P* < 0.001), AST (β-coefficient = − 0.08; 95% CI: − 0.12 to − 0.03; *P* = 0.003), ALT (β-coefficient = − 0.05; 95% CI: − 0.10 to − 0.001; *P* = 0.047), ALP (β-coefficient = − 0.02; 95% CI: − 0.04 to − 0.01; *P* = 0.01), TB (β-coefficient = − 1.64; 95% CI: − 2.83 to − 0.44; *P* = 0.008), as well as DB (β-coefficient = − 1.68; 95% CI: − 2.92 to − 0.43; *P* = 0.009) and a deline in platelet number (β-coefficient = 9.04 × 10^–5^; 95% CI: 0.00 to 0.00; *P* = 0.002) (Table 2).

**Table 2:** Spearman's rho correlation and multivariate linear regression analyses of circulating CLU estimates.

Variables	Circulating CLU levels (μg/mL)			
	Spearman's rho correlation		Linear regression^a^	
	Coefficient (*r*)	*P*-value	β coefficient (95% CI)	*P*-value
Gender (male:female)	0.12	0.24	–	–
Age (years)	0.11	0.28	–	–
BMI (kg/m^2^)	0.29	0.09	–	–
Liver stiffness (kPa)	− 0.54	< 0.001*	− 0.50 (− 0.71 to − 0.30)	< 0.001*
AST (IU/L)	− 0.44	< 0.001*	− 0.08 (− 0.12 to − 0.03)	0.003*
ALT (IU/L)	− 0.39	< 0.001*	− 0.05 (− 0.10 to − 0.001)	0.047*
ALP (IU/L)	− 0.37	< 0.001*	− 0.02 (− 0.04 to − 0.01)	0.01*
Albumin (g/dL)	0.42	< 0.001*	4.45 (− 2.22 to 11.13)	0.19
TB (mg/dL)	− 0.45	< 0.001*	− 1.64 (− 2.83 to − 0.44)	0.008*
DB (mg/dL)	− 0.55	< 0.001*	− 1.68 (− 2.92 to − 0.43)	0.009*
Platelet (cells/m^3^)	0.31	0.013*	9.04 × 10^–5^ (0.00 to 0.00)	0.002*
PT (s)	− 0.17	0.20	–	–
PTT (s)	− 0.24	0.061	–	–
APRI	− 0.46	< 0.001*	0.03 (− 1.58 to 2.12)	0.77
INR	− 0.27	0.035*	− 27.65 (− 75.00 to 19.70)	0.25

*ALP* alkaline phosphatase, *ALT* alanine aminotransferase, *APRI* AST to platelet ratio index, *AST* aspartate aminotransferase, *BA* biliary atresia, *BMI* body mass index, *DB* direct bilirubin, *INR* international normalized ratio, *PT* prothrombin time, *PTT* partial thromboplastin time, *TB* total bilirubin, *NA* not available.

*Correlation is considered statistically significant at *P*-value less than 0.05 (two-tailed).

^a^The coefficient was adjusted for gender, age, and BMI.

### Relationship between circulating CLU levels and survival rates of BA patients

**Figure 2:**
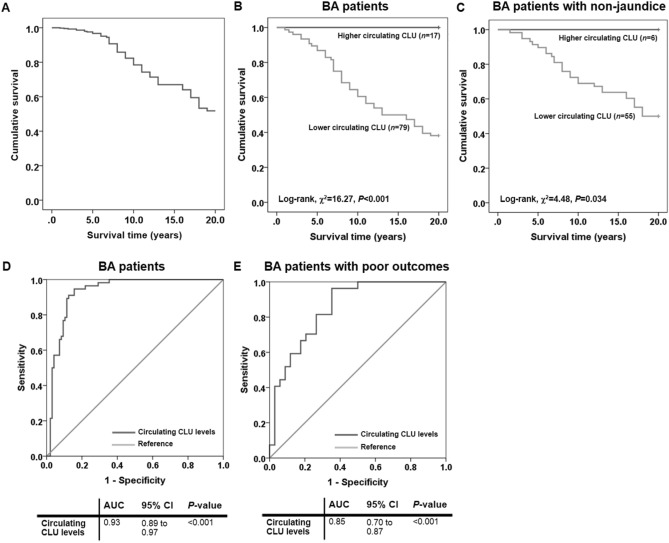
Kaplan–Meier survival curve of post-operative BA patients over 20 years and receiver operating characteristic curve demonstrating diagnostic value of circulating CLU in BA patients. **(A)** Overall survival curve of BA patients. **(B)** Survival curve comparison in BA patients with lower and higher circulating levels of CLU. **(C)** Survival curve comparison in BA patients without jaundice along with lower and higher circulating levels of CLU. **(D)** Circulating CLU levels as a biomarker for distinguishing BA patients from healthy controls. **(E)** Circulating CLU levels as a biomarker for discriminating BA patients with poor outcomes from good outcomes.

Owing to the relevance of circulating CLU levels to poor outcomes in post-Kasai BA patients, Kaplan–Meier analysis was performed to evaluate the effect of lower circulating CLU levels on poor survival of the patients. Figure 2A shows that overall 20-year survival rate for BA patients after HPE was 50.0%. When categorized into lower- (*n* = 79) and higher (*n* = 17) circulating CLU levels based on its median distribution in healthy controls with the cut-off value of 155.26 μg/mL, using log-rank test, the 20-year survival rate of BA patients with lower circulating CLU levels (47.2%) was significantly lower than that of the patients with higher circulating CLU levels (100.0%) (χ^2^ = 16.27, *P* < 0.001), as shown in Fig. 2B. In survival analysis stratified by their jaundice status, lower circulating CLU levels (*n* = 55) were found to be remarkably associated with poor survival of BA patients with non-jaundice, compared with those with higher circulating CLU levels (*n* = 6) (47.3% vs. 100.0%, χ^2^ = 4.48, *P* = 0.034), as depicted in Fig. 2C.

### Circulating CLU levels as a possible biomarker of BA

To identify whether circulating CLU could be employed as a non-invasive biomarker for BA development, the AUC of the ROC curve was constructed using circulating CLU values. The ROC curve analysis illustrated that the optimal cutoff value of circulating CLU as a useful biomarker for discriminating BA patients from healthy controls was projected to be 129.01 μg/mL, which yielded a sensitivity of 94.6%, a specificity of 84.4%, and an AUC of 0.93 (95% CI: 0.89 to 0.97; *P* < 0.001) (Fig. 2D).

The possibility whether circulating CLU levels allow distinguishing post-operative BA patients with poor outcomes from those with good outcomes was additionally tested. Based on the ROC curve, the optimal cutoff value of circulating CLU was defined at 115.79 μg/mL, and the AUC was 0.85 (95% CI: 0.76 to 0.95; *P* < 0.001). Sensitivity and specificity of circulating CLU as a possible biomarker for poor outcomes of post-Kasai BA patients were 81.5% and 73.5%, respectively (Fig. 2E).

## Discussion

As CLU acts as an ATP-independent chaperon protein associated with the clearance of cellular debris and apoptosis from ECM^6^, in which the imbalance between synthesis and degradation of ECM components contributes to fibrogenesis^16^, it is unsurprising that CLU has been hypothesized to be a biologically active mediator for liver fibrogenesis. Supporting this assumption, a number of previous studies showed its significant involvement in many aspects of cholestatic liver diseases – especially liver fibrosis^13,17,18^. Specifically, Aigelsreiter et al.^15^ immunohistochemically investigated hepatic CLU expression in cholestatic liver disease in the context of liver fibrosis, cirrhosis, as well as HCC and reported that CLU overexpression was associated with accumulation of elastic fibers in patients with liver fibrosis and cirrhosis. Besides, a study by Janig et al.^19^ unveiled CLU overexpression in the portal tracts of human livers elastic fibers, and its expression was associated with altered elastic fibers, in which formation of elastic fibers was reportedly detected in areas of fibrous septa and related to development of liver fibrosis in chronic liver diseases^16,20^. Since CLU overexpression has been extensively delineated in the elastic fibers involved in liver fibrogenesis in response to chronic liver injury, whether systemic production of CLU is associated with severity of liver fibrosis remains to be determined. In this context, we evaluated the possible associations between circulating CLU levels and outcome parameters including degree of liver fibrosis of post-Kasai BA patients. Strikingly, main results derived from our study revealed a significant decrement of circulating CLU levels in post-operative BA patients – especially those with jaundice, severe fibrosis, and late stage of hepatic dysfunction. Further analysis uncovered that reduced circulating CLU levels were substantially associated with increased values of liver stiffness, AST, ALT, TB, DB, ALP, and INR, but correlated with declined levels of serum albumin and platelet number in BA patients. All our findings suggest that a decrease in circulating CLU levels may be involved in impartment of hepatic function and development of liver fibrosis in post-operative BA. Although the association of circulating CLU levels with BA severity has as yet not been investigated, there are considerable published data on the systemic and local levels of CLU in patients with chronic multifactorial diseases—particularly cancer patients^21–23^. Amongst others, a case–control study by Wang et al.^24^ measured serum CLU levels in HCC patients and also depicted that HCC patients exhibited considerably lower serum CLU levels than patients with chronic hepatitis B and healthy controls. In contrast to this previous finding, Nafee et al.^25^ reported a marked increase in serum CLU levels in patients with HCC, which was in agreement with a main finding of a recent study by Zheng et al.^26^. The authors remarked that serum CLU levels were significantly elevated in patients with HCC, compared with patients with cirrhosis, chronic hepatitis, and healthy controls. In parallel with this result, CLU expression was found to be remarkably overexpressed in cancerous livers of patients with HCC. Interestingly, changes in serum level and expression of CLU were closely correlated with tumor progression. All above-mentioned findings suggest that circulating CLU may have a diagnostic value as a non-invasive biomarker indicating severe liver complications of patients with chronic liver diseases including BA patients. To address this speculation, we further explored whether circulating CLU may be utilized as a novel indicator for poor outcomes of post-operative BA patients. The ROC curve analysis showed that circulating CLU could be developed as a useful biomarker for discriminating post-operative BA patients with poor outcomes from good outcomes. Given that a diagnostic value of circulating CLU for monitoring poor outcomes in post-operative BA patients derived from ROC curve analysis showed an AUC of less than 0.90, it is important to note that a combination of circulating CLU and existing liver function markers can bring about improvements in the assessment of clinical outcomes in the patients. With regard to a significant decrease in circulating levels of CLU in post-operative BA patients with poor outcomes, we additionally explored the effect of low circulating CLU on survival rates of post-Kasai BA patients and also observed that the patients with lower circulating CLU levels showed significantly reduced survival times, when compared with those with higher circulating CLU levels, suggesting decreased survival in the patients with lower circulating CLU levels. This finding was inconsistent with previous result derived from the aforementioned study of Zheng et al.^26^, which noted that higher CLU expression was significantly associated with poor survival of HCC patients. The exact reason for this conflicting result remains unexplained. It may be attributed to a difference in the pathophysiology of disease between studies. As per an additional role of CLU in immune response, it reportedly inhibited macrophage infiltration and proinflammatory M1 polarization in response to inflammation^27^, which may be a mechanistic explanation of why low circulating CLU levels were associated with poor survival in BA patients given immune dysregulation recognized as one of the pathological events driving BA development. In the light of our considerations, it is tempting to postulate that a decrease in circulating CLU levels in BA patients and those with advanced-stage might reflect a defensive mechanism of the body in response to hepatic impairment and further fibrogenesis. Regardless of its primary action as a chaperon protein responsible for ECM homeostasis, it has been hypothesized that CLU may inhibit precipitation of biliary proteins, possibly resulting in an imbalance between synthesis and degradation of bile acid implicated in progressive cholestasis. This speculated fate may lead to declined circulating CLU levels in the patients. Considering a close link between circulating CLU and liver fibrosis, another possible explanation for reduced circulating CLU in BA patients with severe fibrosis might result from limited extracellular chaperone function of CLU in shielding elastic materials, which in turn would alter production of ECM components participating in fibrogenic process. Collectively, these phenomena may help explain why a decline in circulating CLU levels was found in BA patients, especially in those with jaundice and severe fibrosis and was associated with the disease severity. However, the underlying mechanisms involved in CLU reduction in the circulation of BA patients remain to be elucidated further.

The current study inevitably had some inherent caveats, which need to be taken into account when evaluating the findings presented herein. The most notable drawback is the fact that this study is cross-sectional in design with a relatively small number of participants. In this regard, it is difficult to determine the mechanisms underlying the causal relationships between circulating CLU and BA outcomes. To address this question of cause or consequence, multi-center prospective cohorts with larger sample sizes will help to verify any relationships. Furthermore, we evaluated systemic production of CLU, but did not explore its protein expression in the liver of BA patients. Further immunohistochemical studies of CLU in the specific local tissues would yield more valuable information on its localization in BA livers. Alongside, we were unable to determine the involvement of CLU in inflammatory response in BA patients. To address this latter point, circulating levels of inflammatory cytokines such as interleukin (IL)-1β, IL-6, and tumor necrosis factor-α should be measured in both BA patients with low and high circulating CLU levels. Besides this, due to unavailability of data on co-morbidities of BA makes it difficult to interpret our result about whether low circulating CLU levels were independently associated with reduced survival rates of post-operative BA patients. Additionally, as the study participants are from hospital-based participants rather than the general population, there might be some risk of selection bias if they had any differences in terms of the studied exposures.

Altogether, this is the first study to provide evidence of decreased circulating CLU levels in post-operative BA patients – particularly in those with advanced-stage including jaundice, severe fibrosis, and late-stage of hepatic dysfunction. Furthermore, circulating CLU levels were closely correlated with clinical parameters indicating jaundice status, degree of fibrosis, and liver dysfunction in the patients. Interestingly, reduced circulating CLU levels could predict worse survival of post-operative BA patients. All findings suggest that alterations in circulating CLU levels associated with outcome parameters would reflect severe liver complications of BA patients following Kasai operation and could be a novel biomarker in combination with conventional liver function markers for predicting the disease severity. However, currently available data are derived from in-depth analyses of these associations, in which additional prospective studies are highly desirable to enable more precise estimates and a better understanding of the casual connection of CLU and BA outcomes.

## Abbreviations

ALP: Alkaline phosphatase
ALT: Alanine aminotransferase
ANOVA: One-way analysis of variance
APRI: AST to platelet ratio index
AST: Aspartate aminotransferase
AUC: Area under curve
BA: Biliary atresia
CLU: Clusterin
ELISA: Enzyme-linked immunosorbent assay
HCC: Hepatocellular carcinoma
HPE: Hepatoportoenterostomy
INR: International normalized ratio
PT: Prothrombin time
PTT: Partial thromboplastin time
ROC: Receiver operating characteristic
SD: Standard deviation
TB: Total bilirubin
NA: Not available
